# The Hospital Sunday Fund

**Published:** 1898-09-24

**Authors:** 


### THE HOSPITAL SUNDAY FUND.

We notice in the London Letter of an American contemporary the following comment on the Hospital Sunday Fund: "The Hospital Sunday Fund has a falling off of about £5,000 this year. Various explanations are offered by its supporters, the favourite one being the competition of the Prince of Wales's fund. Sir Sydney Waterlow adopts this notion, but Sir H. Burdett ridicules it. Neither of them seems to perceive that discontent with the decisions and the treatment meted out to deputations has much to do with it. Sir S. Waterlow's prejudices a gainst specialist render his manner of receiving them objectionable, and the secretary of the fund, taking his cue from the chairman, can be most offensive." Is all this true? Is it a fact that the resources of the Fund are lessened by "the treatment meted out to the deputations," that Sir Sydney Waterlow has "prejudices" against specialists, that his manner of receiving them is "objectionable," and that the secretary "can be most offensive"? Let us judge the truth of one part of the paragraph by the truth of the other. The writer of the London Letter says that the Hospital Sunday Fund has a falling off of about £5,000 this year; but on inquiry we are informed that as a fact the falling off is under £2,000, and that there is still money to come in. If the writer of this London Letter is so ill-informed as to facts we hardly need follow him in his fancies about "treatment meted out to deputations," about "prejudices," about "manner," and about one man "taking his cue" from another. It must be remembered that the reason which, above all else, has led the public to trust and support the Hospital Sunday Fund is the knowledge that the money subscribed to it is not lazily handed over to a heterogeneous mass of so-called charities, but that a careful selection is made according to definite rules framed with the intent of encouraging the more worthy among them. Selection of many must of necessity involve the rejection of some, and among these are, no doubt, certain special institutions. But if they want the money let them conform.

